# Disturbances in Calcium Homeostasis Promotes Skeletal Muscle Atrophy: Lessons From Ventilator-Induced Diaphragm Wasting

**DOI:** 10.3389/fphys.2020.615351

**Published:** 2020-12-17

**Authors:** Hayden W. Hyatt, Scott K. Powers

**Affiliations:** Department of Applied Physiology and Kinesiology, University of Florida, Gainesville, FL, United States

**Keywords:** oxidative stress, reactive oxygen species, muscle atrophy, ryanodine receptors, calpain, proteolysis

## Abstract

Mechanical ventilation (MV) is often a life-saving intervention for patients in respiratory failure. Unfortunately, a common and undesired consequence of prolonged MV is the development of diaphragmatic atrophy and contractile dysfunction. This MV-induced diaphragmatic weakness is commonly labeled “ventilator-induced diaphragm dysfunction” (VIDD). VIDD is an important clinical problem because diaphragmatic weakness is a major risk factor for the failure to wean patients from MV; this inability to remove patients from ventilator support results in prolonged hospitalization and increased morbidity and mortality. Although several processes contribute to the development of VIDD, it is clear that oxidative stress leading to the rapid activation of proteases is a primary contributor. While all major proteolytic systems likely contribute to VIDD, emerging evidence reveals that activation of the calcium-activated protease calpain plays a required role. This review highlights the signaling pathways leading to VIDD with a focus on the cellular events that promote increased cytosolic calcium levels and the subsequent activation of calpain within diaphragm muscle fibers. In particular, we discuss the emerging evidence that increased mitochondrial production of reactive oxygen species promotes oxidation of the ryanodine receptor/calcium release channel, resulting in calcium release from the sarcoplasmic reticulum, accelerated proteolysis, and VIDD. We conclude with a discussion of important and unanswered questions associated with disturbances in calcium homeostasis in diaphragm muscle fibers during prolonged MV.

## Introduction

Mechanical ventilation (MV) is often a life-saving intervention for both critically ill patients and patients undergoing surgery. An unwanted side-effect of prolonged MV is the rapid development of inspiratory muscle weakness that occurs due to both diaphragmatic atrophy and contractile dysfunction. Collectively, this syndrome has been labeled ventilator-induced diaphragm dysfunction (VIDD) (Vassilakopoulos and Petrof, 2004). VIDD is a serious clinical problem because diaphragmatic weakness is a major risk factor contributing to the failure to wean patients from the ventilator (Petrof et al., 2010).

Abundant evidence confirms that MV-induced diaphragmatic atrophy occurs due to both a decrease in muscle protein synthesis and increased proteolysis with proteolysis playing a dominant role (Whidden et al., 1985b; Shanely et al., 2002, 2004; Agten et al., 2011; Powers et al., 2013; Smuder et al., 2014, 2018; Hudson et al., 2015). The MV-induced increase in proteolysis within diaphragm fibers is triggered by increases in mitochondrial production of reactive oxygen species (ROS); this redox imbalance contributes to the activation of the four major proteolytic systems in skeletal muscle (i.e., ubiquitin-proteasome, autophagy, calpain, and caspase-3) (Powers et al., 2011). Although all of these proteolytic systems contribute to MV-induced diaphragmatic atrophy, activation of the calcium (Ca^2+^)-activated protease, calpain, plays a central role in MV-induced diaphragmatic atrophy. Indeed, blockade of calpain activation in the diaphragm can markedly reduce both MV-induced diaphragmatic atrophy and contractile dysfunction (Maes et al., 2007; Nelson et al., 2012).

This review provides a summary of the cell signaling events leading to VIDD with a focus on the cellular processes that result in disturbed Ca^2+^ homeostasis and the subsequent activation of calpain within diaphragm muscle fibers. More specifically, we discuss the evidence that increased mitochondrial production of ROS results in modification of the ryanodine receptor/Ca^2+^ release channel in the sarcoplasmic reticulum (SR), resulting in the release of Ca^2+^ from the SR, calpain activation, and VIDD. In an effort to stimulate future research, we also highlight unanswered questions associated with signaling events.

## The Problem: Ventilator-Induced Diaphragmatic Weakness

The observation that prolonged MV results in diaphragmatic wasting was first reported in a retrospective study revealing that diaphragmatic atrophy was present in infants and neonates exposed to prolonged MV (Knisely et al., 1988). Direct evidence to support this conjecture was later provided by a preclinical study revealing that 48 h of MV results in marked diaphragmatic atrophy and contractile dysfunction (Le Bourdelles et al., 1996). Since these initial reports, numerous studies have confirmed that as few as 12–24 h of MV results in VIDD in both animals and humans [reviewed in Powers et al. (2013)].

Two primary modes of MV exist: (1) partial support and (2) full support MV. During partial support MV, the ventilator assists during inspiration, but the patients’ inspiratory muscles remain engaged in breathing. During full support MV, the ventilator performs all of the work of breathing, resulting in inactivity of the diaphragm and other inspiratory muscles; compared to partial support, full support MV results in a more rapid rate of VIDD. Indeed, when contrasted with inactivity-induced limb muscle atrophy (e.g., prolonged bedrest), full support MV-induced diaphragmatic atrophy is a unique form of skeletal muscle atrophy that occurs extremely rapidly after the onset of MV. For instance, the cross-sectional area (CSA) of diaphragm muscle fibers is reduced by >15% within the first 12–18 h of MV in both rats and humans (Whidden et al., 1985a; Shanely et al., 2002; Levine et al., 2008; Nelson et al., 2012). In comparison with inactivity-induced atrophy in limb muscles, 5–7 days of inactivity would be required to achieve this magnitude of fiber atrophy in locomotor skeletal muscles (Powers et al., 2013). In this regard, the diaphragm muscle differs from limb muscle in several aspects. First, the diaphragm is chronically active, contracting several times per minute even during sleep (Lessa et al., 2016; Fogarty et al., 2018). Moreover, the diaphragm also contributes to several non-respiratory activities including swallowing and vocalization (Fogarty et al., 2018). Further, limb skeletal muscles exert force along the longitudinal axis of the fiber because diaphragm fibers are exposed to a pressure load both longitudinally and perpendicularly to the axis of the muscle (Lessa et al., 2016).

As mentioned previously, the diaphragmatic weakness associated with VIDD is a primary risk factor for the failure to wean patients from the ventilator. In this context, weaning is defined as the ability to remove patients from ventilator support and restore spontaneous breathing. The incidence of difficult weaning of patients from MV is variable but can reach >30% or higher in those ventilated for more than 3 days (Funk et al., 2010). The inability to wean patients from MV is a serious clinical problem that results in prolonged stays in the intensive care unit and significant increases in both morbidity and mortality (Funk et al., 2010). Developing a therapy to prevent VIDD and reduce the risk of weaning problems requires a thorough understanding of the signaling events that promote VIDD. The next segments provide a summary of our current knowledge of the cellular processes that promote MV-induced diaphragmatic weakness.

## MV-Induced Diaphragmatic Atrophy: Proteolysis vs Depressed Protein Synthesis

The size of skeletal muscle fibers is controlled by the relative balance between rates of protein synthesis and protein breakdown. It is well established that prolonged MV results in a rapid increase in proteolysis and a decline in protein synthesis in the rodent diaphragm (Shanely et al., 2004). In regard to the rates of diaphragmatic protein synthesis, both mixed and myofibrillar protein synthesis rates decline within the first 6 h of MV (Shanely et al., 2004). While this MV-induced depression in protein synthesis rates clearly contributes to diaphragmatic atrophy during prolonged MV (i.e., days to weeks), the rapid atrophy that occurs in the human or rat diaphragm (i.e., >15% reduction in diaphragm CSA) within the first 12–24 h of MV is largely driven by a swift increase in proteolysis (Powers et al., 2013). In reference to the proteolytic systems that contribute to MV-induced diaphragm atrophy, abundant evidence indicates that all four of the major proteolytic systems in skeletal muscle (i.e., calpains, caspase-3, ubiquitin-proteasome system, and autophagy) are active during prolonged MV (DeRuisseau et al., 1985; Kwon et al., 1985; Maes et al., 2007; McClung et al., 2007, 2008; Agten et al., 2011; Nelson et al., 2012; Smuder et al., 2018). Although each of these proteolytic systems contributes to MV-induced diaphragmatic wasting, evidence reveals that the calcium-activated protease calpain plays a key role. Because increases in cytosolic free Ca^2+^ are required to activate calpains, the next segment provides a brief overview of the regulation of cellular Ca^2+^ levels.

## Regulation of Ca^2+^ Homeostasis in Skleletal Muscle

Calcium ions play important roles in several signaling events in skeletal muscle fibers including excitation-contraction (E-C) coupling and a variety of cell signaling pathways. Importantly, high levels of free Ca^2+^ in the cytosol can be toxic to cells by promoting increased mitochondrial ROS production, protease activation, and myonuclear apoptosis (Bogeski et al., 2011; Vallejo-Illarramendi et al., 2014; Rossi et al., 2019). Therefore, it is not surprising that skeletal muscle fibers contain an intricate regulatory system that tightly manages the levels of free Ca^2+^ in the fiber. Indeed, Ca^2+^ concentration is controlled in muscle fibers via a network of voltage sensors, Ca^2+^ transporters, Ca^2+^ channels, Na^+^/Ca^2+^ exchangers, Ca^2+^-binding proteins, and ion pumps/exchangers. A detailed discussion of the regulation of Ca^2+^ homeostasis is beyond the scope of this review, and the reader is directed to comprehensive reviews on this topic for more details (Berridge et al., 2003; Bogeski et al., 2011; Vallejo-Illarramendi et al., 2014; Belosludtsev et al., 2019; Rossi et al., 2019). Nevertheless, prior to a discussion of the mechanisms responsible for MV-induced disturbances in Ca^2+^ homeostasis in diaphragm fibers, a brief introduction to some of the key checkpoints in cellular Ca^2+^ homeostasis is provided in the next segments.

### Sarcolemma Regulation of Ca^2+^ Influx/Efflux

**FIGURE 1:**
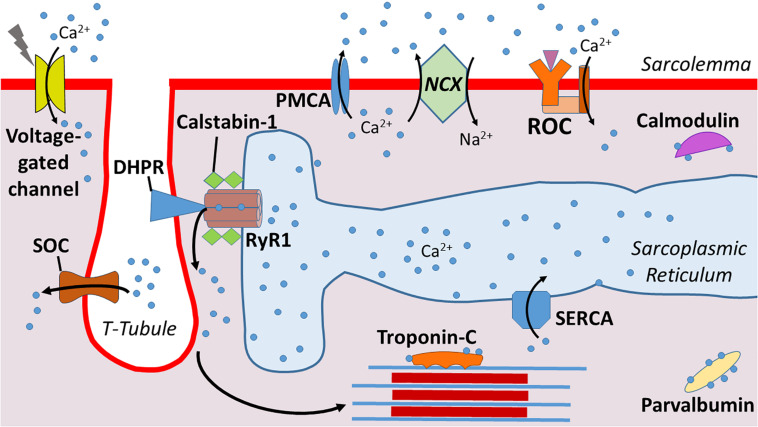
Illustration of calcium (Ca^2+^) regulation at the sarcolemma. Dihydropyridine receptors (DHPR) mediate Ca^2+^ release from the sarcoplasmic reticulum into the cytosol. Calstabin-1 is bound to RyR1 and stabilizes this complex to aid in maintaining a closed channel. Ca^2+^ is pumped back into the sarcoplasmic reticulum via sarcoplasmic reticulum Ca^2+^-ATPase (SERCA). Ca^2+^ enters from the extracellular space via voltage-gated channels, store-operated channels (SOC), and receptor operated channels (ROC). Ca^2+^ is excreted from the cytosol via plasma membrane Ca^2+^ ATPases (PMCA) and Na^+^/Ca^2+^ exchanger (NCX). Levels of cytosolic free Ca^2+^ are also regulated by Ca^2+^ binding proteins such as Troponin-C, parvalbumin, and calmodulin.

By design, the sarcolemma has a low permeability for Ca^2+^ as evidenced by the large difference (∼10,000-fold) between the extracellular and cytosolic Ca^2+^ concentration (Vallejo-Illarramendi et al., 2014). Because of this significant concentration gradient, Ca^2+^ movement from the extracellular fluid into the cytosol of muscle fibers is driven by the large electrochemical gradient across the sarcolemma. The major influx pathways for Ca^2+^ entry into the cytosol include voltage-gated channels, receptor operated channels (ROC), and store operated entry channels (SOC) (Vallejo-Illarramendi et al., 2014; Figure 1).

Because high cytosolic Ca^2+^ levels are toxic to muscle fibers, the sarcolemma is equipped with ATPases – e.g., plasma membrane Ca^2+^ ATPases (PMCA) – to pump Ca^2+^ from the cytosol across the sarcolemma (DeSantiago et al., 2007). Further, the Na^+^/Ca^2+^ exchanger (NCX) also contributes to Ca^2+^ exchange across the sarcolemma (Burr et al., 2014). Compared to the PMCA, the NCX has a greater Ca^2+^ transport rate and therefore, can protect against Ca^2+^ overload in the cytosol over a wider range of Ca^2+^ transients (Brini and Carafoli, 2011). In healthy muscle fibers, the combined properties of these Ca^2+^ transport proteins are effective in maintaining cytosolic Ca^2+^ homeostasis.

### Cytosolic Ca^2+^ Buffers

Cytosolic Ca^2+^ buffers are Ca^2+^ binding proteins that provide another means of regulating cytosolic levels of free Ca^2+^. Indeed, the rapid binding of Ca^2+^ to cytosolic buffers make Ca^2+^ binding molecules an ideal strategy to regulate free Ca^2+^ levels in the cytosol (Schwaller, 2010). Important Ca^2+^ binding proteins in skeletal muscle fibers include troponin C, parvalbumin, and calmodulin (Kretsinger, 1976). Collectively, these Ca^2+^ binding proteins compose a network of molecules that modulate signaling (Yanez et al., 2012).

### Ca^2+^-Handling Within the Sarcoplasmic Reticulum

The SR, a highly specialized type of endoplasmic reticulum, is the primary reservoir for Ca^2+^ storage in muscle fibers (Pietrobon et al., 1990; Vallejo-Illarramendi et al., 2014). The SR is an organized tubular network that surrounds myofibrils with a dilated end sac labeled as the terminal cisternae (Figure 1). An extension of the sarcolemma forms the central T-tubule responsible for penetrating into the muscle cell and facilitating Ca^2+^ release. This structure is flanked on each side by terminal cisternae that form a region within the fiber called the triad; the triad serves as the anatomical structure responsible for triggering excitation-contraction coupling in skeletal muscle fibers.

The SR plays an essential role in the management of intracellular Ca^2+^ within skeletal muscle fibers. In particular, three major categories of Ca^2+^ handling proteins exist within the SR: (1) SR Ca^2+^ release channels; (2) SR Ca^2+^ binding proteins; and (3) Ca^2+^ ATPase pumps. A brief summary of these classes of SR handling proteins follows.

The primary Ca^2+^ release channel in the SR is the ryanodine receptor (RyR). Three isoforms of the RyR (i.e., RyR1, RyR2, and RyR3) exist in mammals, but RyR1 is the dominant isoform expressed in adult skeletal muscle and is crucial for muscular contraction (Capes et al., 2011; Vallejo-Illarramendi et al., 2014). RyR1 is the largest ion channel known (∼2.3 MDa) and is composed of four identical subunits; this organization forms a pore within the SR membrane that serves as the Ca^2+^ release channel (Hernandez-Ochoa et al., 2015). Importantly, the subunit structure of RyR1 is associated with several accessory proteins including four calstabin-1 subunits (also known as FK506 binding protein 1A) (Wehrens et al., 2005). Calstabin is a key molecule regulating RyR1 function because, in a resting (non-contracting muscle), the binding of calstabin to RyR1 maintains the channel in a closed state to prevent leakage of Ca^2+^ from the SR into the cytoplasm (Brillantes et al., 1994).

Upon depolarization of the sarcolemma, the opening of the RyR1 is regulated by dihydropyridine receptors (DHPRs) (Capes et al., 2011; Vallejo-Illarramendi et al., 2014). DHPRs are mechanically linked to RyR1, and the voltage change induced by depolarization of the sarcolemma results in a conformational change in DHPR that opens the RyR1 (Protasi et al., 2002). This activation and opening of the RyR1 releases Ca^2+^ from the SR into the cytosol of muscle fibers, triggering E-C coupling and increased muscle force production. Notably, the magnitude of Ca^2+^ release from the SR into the cytosol is a primary determinant of the amount of force generated during muscular contraction (Wehrens et al., 2005).

In addition to the modulatory effects of DHPRs and calstabin-1, Ca^2+^ release through the RyR1 is also regulated by several post-translational events along with numerous proteins and small molecules. Cytosolic regulators of RyR1 include ATP, calmodulin II, and protein kinase A (PKA) (Vallejo-Illarramendi et al., 2014). RyR1 activity can also be regulated by oxidative stress, as oxidative modification of cysteine thiol residues such as S-nitrosylation, S-glutathionylation, and disulfide oxidation have been shown to modulate RyR1 function. In particular, oxidation, coupled with PKA-induced phosphorylation of RyR1, decreases the binding association between calstabin-1 and the RyR1 complex; this results in a leak of Ca^2+^ from the SR into the cytosol (Reiken et al., 2003; Ward et al., 2003).

### Mitochondrial Ca^2+^ Handling

Along with their key role in muscle bioenergetics, mitochondria can also take up and store Ca^2+^ transiently (Rossi et al., 2019). Two subpopulations of mitochondria exist in skeletal muscle: (1) subsarcolemmal and (2) intermyofibrillar. As the names imply, subsarcolemmal mitochondria are located directly beneath the sarcolemma whereas intermyofibrillar mitochondria are found surrounding the myofibrillar contractile proteins. The adjacent locations of the subsarcolemmal mitochondria and the SR allow for significant interaction between these organelles; indeed, areas of adjacent SR and mitochondria form microdomains of contact that are described as mitochondria associated membranes (MAMs). MAMs describe the areas where SR proteins directly associate with components of the outer mitochondrial membrane (Csordas et al., 2006). This linkage facilitates efficient Ca^2+^ transfer between these two organelles and therefore, mitochondria actively contribute to Ca^2+^ homeostasis in skeletal muscles.

**FIGURE 2:**
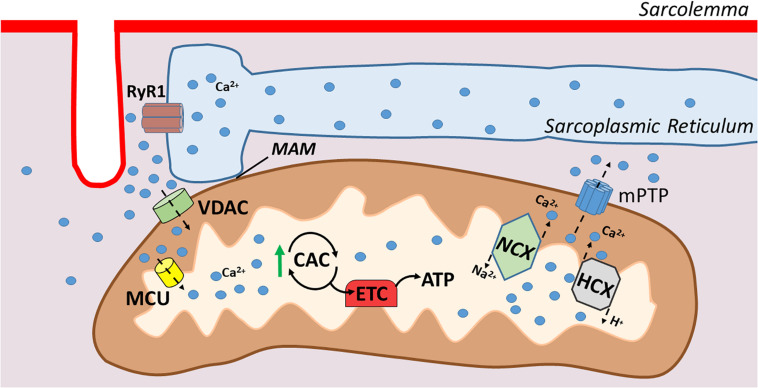
Illustration of mitochondrial calcium transport. Mitochondrial associated membranes (MAMs) result in microdomains of high Ca^2+^ levels that facilitate Ca^2+^ entry through the outer mitochondrial membrane via voltage dependent anion channel (VDAC). Ca^2+^ crosses the inner mitochondrial membrane via the mitochondrial uniporter (MCU), which then stimulates respiration via increases in citric acid cycle enzymes. Ca^2+^ is extruded from the mitochondria via Na^+^/Ca^2+^ exchanger (NCX) and H^+^/Ca^2+^ exchanger (HCX) in the inner membrane and the outer membrane via the mitochondrial permeability transition pore (mPTP).

The cytosolic Ca^2+^ fluxes that occur during muscular contraction result in a transient uptake of Ca^2+^ into the mitochondria (Rossi et al., 2019). This Ca^2+^ uptake at the outer mitochondrial membrane is facilitated by the voltage-dependent anion channel (VDAC), which allows for the exchange of molecules and ions between the cytosol and mitochondria. However, the mitochondrial inner membrane is impermeable to ions, and therefore, highly specialized channels are required for Ca^2+^ ions to enter the mitochondrial matrix (Vallejo-Illarramendi et al., 2014; Rossi et al., 2019). Ca^2+^ transport into the mitochondrial matrix is primarily facilitated by the mitochondrial uniporter (MCU) (Vallejo-Illarramendi et al., 2014; Belosludtsev et al., 2019; Rossi et al., 2019; Figure 2). This ruthenium-red-sensitive uniporter utilizes the negative membrane potential across the inner mitochondrial membrane to facilitate Ca^2+^ entry into the mitochondrial matrix. In particular, MAMs form junctures that facilitate microdomains of high Ca^2+^ levels due to their positioning near the SR, and these high Ca^2+^ levels induce the opening of the MCU pore, permitting Ca^2+^ entry into the matrix (Vallejo-Illarramendi et al., 2014; Rossi et al., 2019).

The discovery that mitochondria are capable of Ca^2+^ uptake led to studies revealing that Ca^2+^ is an important regulator of oxidative phosphorylation. For example, Ca^2+^ is a positive allosteric activator for several key enzymes that influence flux through the citric acid cycle (e.g., pyruvate dehydrogenase, isocitrate dehydrogenase, and 2-oxoglutarate dehydrogenase) (Denton et al., 1980; McCormack and Denton, 1980). Moreover, recent evidence shows that Ca^2+^ activates the entire oxidative phosphorylation cascade in skeletal muscle mitochondria (Glancy et al., 2013). In contrast to these metabolically beneficial effects of moderate Ca^2+^ uptake in mitochondria, excessive Ca^2+^ uptake resulting in a Ca^2+^ overload elevates mitochondrial ROS production and increases mitochondrial sensitivity to apoptotic stimuli (Pinton et al., 2008).

The primary means for extrusion of Ca^2+^ from the mitochondria in skeletal muscle is the Na^+^/Ca^2+^ exchanger and the H^+^/Ca^2+^ exchanger located within the inner mitochondrial membrane (Pizzo et al., 2012). Further, during Ca^2+^ overload in the mitochondria, a large conductance channel known as the mitochondrial permeability transition pore (mPTP) can be formed and operates as a conduit for release (Vallejo-Illarramendi et al., 2014). Nonetheless, at present, details about the role that the mPTP plays in regulating mitochondrial Ca^2+^ levels remains poorly understood (Giorgio et al., 2018).

## Disturbances in Diaphragmatic Ca^2+^ Homeostasis Is Required for VIDD

Shanely et al. (2002) provided the earliest evidence that prolonged MV results in disturbances of Ca^2+^ homeostasis in rat diaphragm fibers. Although this study did not directly measure cytosolic Ca^2+^ levels, the results revealed that prolonged MV activates calpain in diaphragm fibers and increased cytosolic levels of free Ca^2+^ is a requirement for calpain activation (Goll et al., 2003). MV-induced activation of calpains in diaphragm fibers has since been confirmed in many studies involving both humans and animals (Whidden et al., 1985b; Maes et al., 2007; Levine et al., 2008; McClung et al., 2008; Nelson et al., 2012; Dridi et al., 2020a). Moreover, two independent studies using calpain inhibitors have demonstrated that calpain is a major contributor to VIDD (Maes et al., 2007; Nelson et al., 2012). A brief overview of calpains and their activation in skeletal muscle follows.

Calpains are a family of Ca^2+^ activated proteases that cleave target substrates at specific sites (Goll et al., 2003). Three primary calpain isoforms exist in skeletal muscle (i.e., calpain1, calpain2, and calpain3); although evidence reveals that calpains 1 and 2 play an important role in promoting disuse muscle atrophy, it remains unknown as to whether calpain1 or calpain2 plays the dominant role in promoting fiber atrophy (Hyatt and Powers, 2020).

While activation of calpain involves several post-translational modifiers, it is established that calpain activation in muscle fibers requires increased cytosolic levels of free Ca^2+^. Though the intracellular concentrations of free Ca^2+^ required to activate calpains in skeletal muscle *in vivo* remain unknown, it is clear that the proteolytic removal of the N-terminal domain of the calpain molecule allows for both calpain1 and calpain2 to be activated at significantly lower Ca^2+^ concentrations (Goll et al., 2003). For a detailed discussion of the factors regulating the activity of calpains see the classic review of Goll et al. (2003) and the recent review by Hyatt and Powers (2020).

To determine the source of increased free Ca^2+^ in the diaphragm during prolonged MV, Talbert et al. (2016) tested the hypothesis that leakage of Ca^2+^ from the RyR1 is required for MV-induced calpain activation in the diaphragm. Cause and effect were determined by treating ventilated animals with the RyR1 blocker, azumolene. Their results revealed that while azumolene did block stimulation-induced Ca^2+^ release from the RyR1, treatment with this RyR1 channel blocker was not sufficient to prevent MV-induced calpain activation or VIDD (Talbert et al., 2016). However, azumolene may not prevent all forms of Ca^2+^ release from RyR1s. For example, while it is established that azumolene prevents neural stimulation-induced Ca^2+^ release from the RyR1, it remains unknown if this compound blocks oxidation-induced release of Ca^2+^ from the RyR1. Hence, experiments using a different investigative approach are required to delineate the role that leaky RyR1s play in MV-induced disturbances in Ca^2+^ homeostasis in diaphragm fibers.

Matecki et al. (2016) performed the first study to directly evaluate the impact of prolonged MV on Ca^2+^ homeostasis in the diaphragm. This elegant study provided robust evidence that prolonged MV increases the opening of RyR1 in diaphragm muscle fibers (Matecki et al., 2016); this increased spontaneous opening of RyR1 results in increased Ca^2+^ release (i.e., Ca^2+^ sparks) and disrupts Ca^2+^ homeostasis (Matecki et al., 2016). Analysis of both human and mouse diaphragm fibers has established that prolonged MV results in oxidation, S-nitrosylation, and Ser-2844 phosphorylation of the RyR1. Importantly, these post-translational modifications of the RyR1 accompany the dissociation of calstabin1 (Matecki et al., 2016). The importance of calstabin-1 association with RyR1 for preventing RyR1 leak was confirmed by findings that treatment of animals with S107, a small molecule that stabilizes the RyR1-calstabin1 interaction, prevents MV-induced Ca^2+^ sparks in diaphragm fibers, and protects against VIDD (Matecki et al., 2016). Furthermore, animals treated with the antioxidant trolox do not experience MV-induced oxidation of RyR1 and RyR1-mediated Ca^2+^ leak in diaphragm fibers. Therefore, this landmark study demonstrates that RyR1 dysfunction is an early pathological event leading to VIDD in both mice and humans, and confirms that oxidative stress is a prerequisite for MV-induced RyR1 dysfunction (Matecki et al., 2016).

**FIGURE 3:**
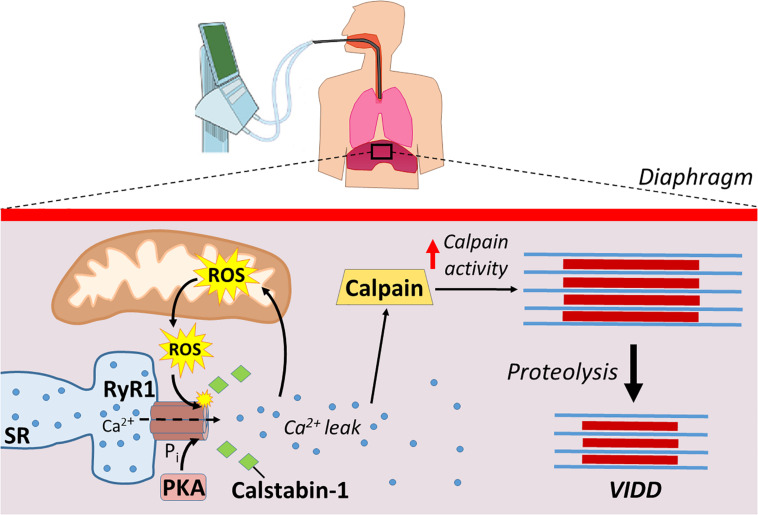
Mechanical ventilation causes Ca^2+^ disruptions that lead to increased proteolysis. ROS production from mitochondria, along with increased protein kinase A (PKA) activity, modify ryanodine receptors (RyR1) leading to the disassociation of calstabin-1. Disassociation of calstabin-1 from RyR1 leads to Ca^2+^ leak, activation of calpains, and increased proteolysis that contributes to VIDD.

While the MV-induced increases in ROS production in the diaphragm is derived from several sources, mitochondrial ROS production is the dominant site of ROS emission in diaphragm fibers during prolonged MV (Falk et al., 1985, 2011; Whidden et al., 1985a; McClung et al., 2009; Powers et al., 2011). Recently, this supposition has gained additional support from a study concluding that MV-induced mitochondrial ROS production in the diaphragm is responsible for the oxidative remodeling of the RyR1 (Dridi et al., 2020b). Specifically, using the mitochondrial-targeted antioxidant SS-31 to prevent MV-induced increases in mitochondrial ROS production, Dridi et al. (2020a) provide further evidence that mitochondrial oxidative stress is responsible for oxidation of RyR1s resulting in SR Ca^2+^ leak, calpain activation, and VIDD (Figure 3). Moreover, prolonged MV is also associated with increased PKA activity, which is posited to play a role in the hyperphosphorylation of the RyR1 within diaphragm fibers (Dridi et al., 2020b). This MV-induced increase in PKA activity and hyperphosphorylation of RyR1 was abolished by treatment of animals with the mitochondrial-targeted antioxidant (SS-31). These findings are consistent with reports that oxidative stress increases PKA activity and decreases the activity of several phosphatases (Brennan et al., 2006; Steinberg, 2013). Collectively, these studies demonstrate that mitochondrial ROS emissions are key in the disruptions of Ca^2+^ homeostasis.

Investigations into the signaling pathways responsible for the MV-induced increases in mitochondrial ROS emission in the diaphragm have provided increased insight into the pathologic events underlying VIDD. Specifically, a growing number of studies suggest that MV-induced mitochondrial dysfunction is controlled by several interacting events including mechanical stimulation of angiotensin II type 1 receptors, along with activation of several signaling molecules including FoxO, STAT3, and Smad3 (Kwon et al., 1985; Smith et al., 2014; Smuder et al., 2015; Tang et al., 2015, 2017). Further, emerging evidence also suggests that accelerated autophagy and increased cytosolic levels of free Ca^2+^ are potential modulators of mitochondrial ROS production, acting as a positive feedback loop (Smuder et al., 2018; Tang and Shrager, 2018). Despite these recent advances in our knowledge of the signaling network responsible for MV-induced increases in mitochondrial ROS production, numerous questions remain unanswered about the pathways responsible for MV-induced mitochondrial dysfunction.

## Discussion

Mechanical ventilation is a life-saving clinical intervention used to provide adequate alveolar ventilation in patients that are incapable of doing so on their own. Common applications of MV include patients suffering from chronic obstructive pulmonary disease, heart failure, acute myocardial infarction, and other critical illnesses. While MV is a life-saving intervention for many patients, prolonged MV promotes the rapid development of VIDD. VIDD is clinically significant because diaphragmatic weakness is a key risk factor contributing to problems in weaning patients from the ventilator. The inability to wean patients from the ventilator results in prolonged hospitalization and increased morbidity and mortality.

Mechanical ventilation-induced diaphragmatic atrophy results from both decreased protein synthesis and accelerated proteolysis. However, MV-induced activation of proteases in the diaphragm play a dominant role in the development of VIDD. Although all major proteolytic systems contribute to this MV-induced increase in proteolysis, evidence suggests that activation of the Ca^2+^-activated protease, calpain, plays a required role in this process. The steps leading to MV-induced calpain activation begin with an increase in mitochondrial ROS emission resulting in redox disturbances in diaphragm fibers. This MV-induced oxidative stress in diaphragm fibers results in post-translation modifications of the RyR1 (e.g., phosphorylation and oxidation) leading to the disassociation of calstabin1 from RyR1 and Ca^2+^ leak from the SR. This disturbance in Ca^2+^ homeostasis provides a required stimulus for calpain activation in the diaphragm leading to accelerated proteolysis and VIDD (Figure 3).

Although significant progress has been made toward understanding the mechanisms responsible for the MV-induced disturbance in Ca^2+^ homeostasis in the diaphragm, several important unanswered questions remain. For example, although it is established that an increase in mitochondrial ROS production is a requirement for MV-induced RyR1 leak leading to increased cytosolic levels of free Ca^2+^, the precise mechanisms responsible for this increased oxidant production remain undefined.

Evidence also exists that accelerated autophagy also promotes an increase in mitochondrial ROS emission in the diaphragm during prolonged MV (Smuder et al., 2018). However, the signaling pathways that connect these two events remain unclear.

Finally, it is generally agreed that an uptake of cytosolic Ca^2+^ into mitochondria can produce a mitochondrial Ca^2+^ overload, resulting in significant increases in mitochondria ROS production (Brookes et al., 2004; Peng and Jou, 2010). Nonetheless, it is currently unknown as to whether the MV-induced increases in cytosolic levels of free Ca^2+^ in the diaphragm are sufficient to produce mitochondrial Ca^2+^ overload and stimulate additional mitochondrial ROS production. This is an important issue that warrants future study.

## Author Contributions

HH and SP formulated the outline of the review and contributed to writing and editing of the manuscript. Both authors approved the final submitted version.

## Conflict of Interest

The authors declare that the research was conducted in the absence of any commercial or financial relationships that could be construed as a potential conflict of interest. The reviewer WN declared a past co-authorship with one of the author SP to the handling editor.

## Abbreviations

MV: Mechanical ventilation
VIDD: Ventilator-induced diaphragm dysfunction
ROS: Reactive oxygen species
SR: sarcoplasmic reticulum
CSA: cross-sectional area
EC: excitation-contraction
SOC: store-operated calcium channel
ROC: receptor-operated calcium channel
PMCA: plasma membrane Ca^2+^ ATPases
NCX: Na^+^/Ca^2+^ exchanger
RyR: ryanodine receptor
DHPRs: dihydropyridine receptors
PKA: Protein Kinase A
MAMs: mitochondria associated membranes
VDAC: voltage dependent anion channel
MCU: mitochondrial uniporter.
